# Spitz nevus with unusual dermoscopic figures in an adult female

**DOI:** 10.1002/ccr3.2801

**Published:** 2020-03-24

**Authors:** Hironori Kawana, Hideki Fujita, Daisuke Fujisawa, Madoka Ishii, Miwa Murata, Ayano Kurumatani, Koremasa Hayama, Masaru Tanaka, Tadashi Terui

**Affiliations:** ^1^ Division of Dermatological Science Department of Dermatology Nihon University School of Medicine Tokyo Japan; ^2^ Department of Dermatology Tokyo Women’s Medical University Medical Center East Tokyo Japan

**Keywords:** adult, dermatology, dermoscopy, malignant melanoma, spitz nevus

## Abstract

Spitz nevus is an important differential diagnosis of malignant melanoma, especially in young adults. This case provides a significant information about unusual dermoscopic features of adult Spitz nevus, which may reflect changes over the years.

## Graphical abstract

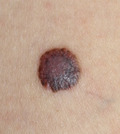

1

A 37‐year‐old Chinese woman was referred to our hospital with a brownish lesion on her right thigh, which had been gradually enlarging for 3 years. On physical examination, a symmetric brown palpable macule with regular borderline, 11‐mm in diameter, was found on the lateral side of her right thigh (Figure 1A). Dermoscopy revealed a central ring‐like bluish‐white area on the pink‐white background surrounded by peripheral brownish structures, which showed typical pigment network (Figure 1B). Streaks or a starburst pattern was absent. We initially suspected this tumor as malignant melanoma and excised it with a 2‐mm surgical margin.

**Figure 1:**
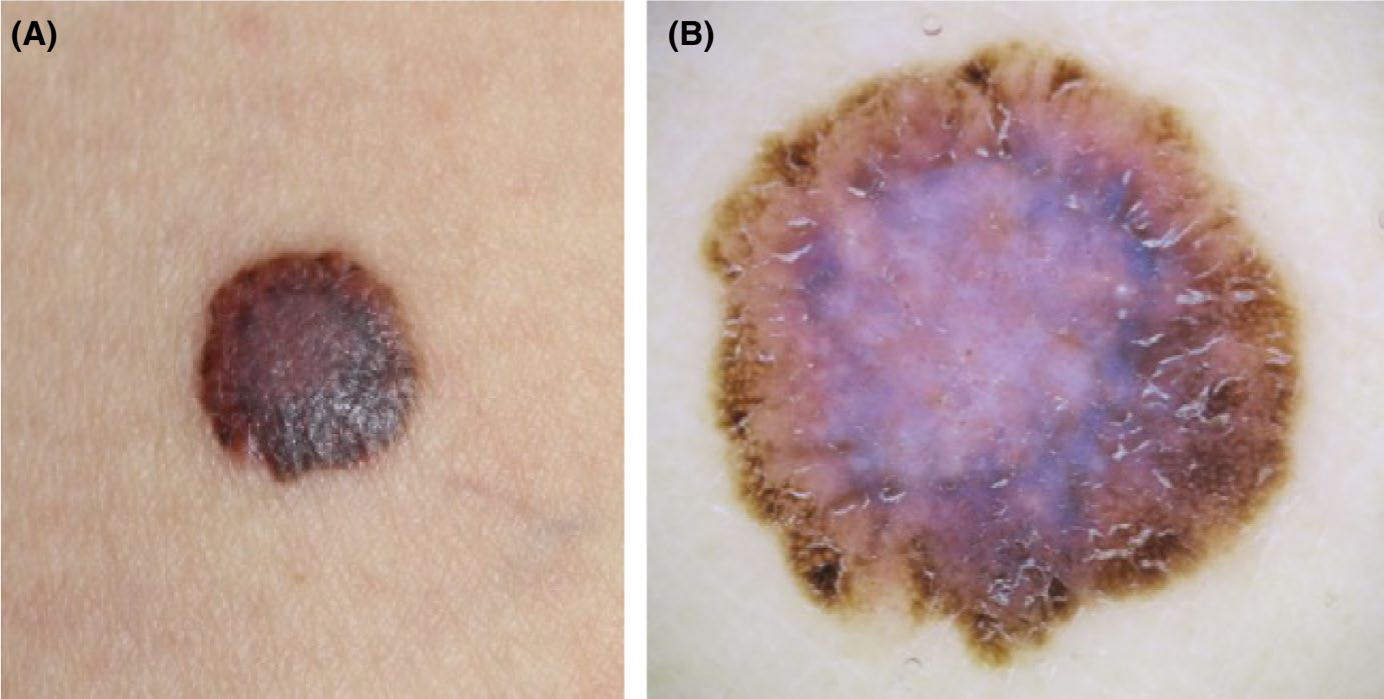
A, A symmetric brown macule, 11 mm in diameter, with regular borderline was present on the lateral side of the right femur. B, A bluish‐whitish area surrounded by a brownish region was observed under dermoscopy. In the brownish region, typical pigmented networks were present

Histopathologically, the lesion showed an inverted triangle‐like form (Figure 2A). Basal hypermelanosis and basilar proliferation of melanocytes were observed on the lateral sides of the lesion (Figure 2B). In the central portion, a small number of atypical melanocytes were observed in the basal layer. There was a proliferation of spindle‐shaped epithelioid melanocytes at the dermoepidermal junction and in the upper dermis, occasionally forming nests (Figure 2C). Melanophages were found around these nests. There was transition from larger nests in the upper dermis to smaller nests and single cells in the lower dermis, indicative of maturation (Figure 2D). Hyperplasia of collagenous fibers and capillary vessels were found around nevus cells (Figure 2E). Kamino bodies were absent. Based on these findings, a diagnosis of Spitz nevus was made.

**Figure 2:**
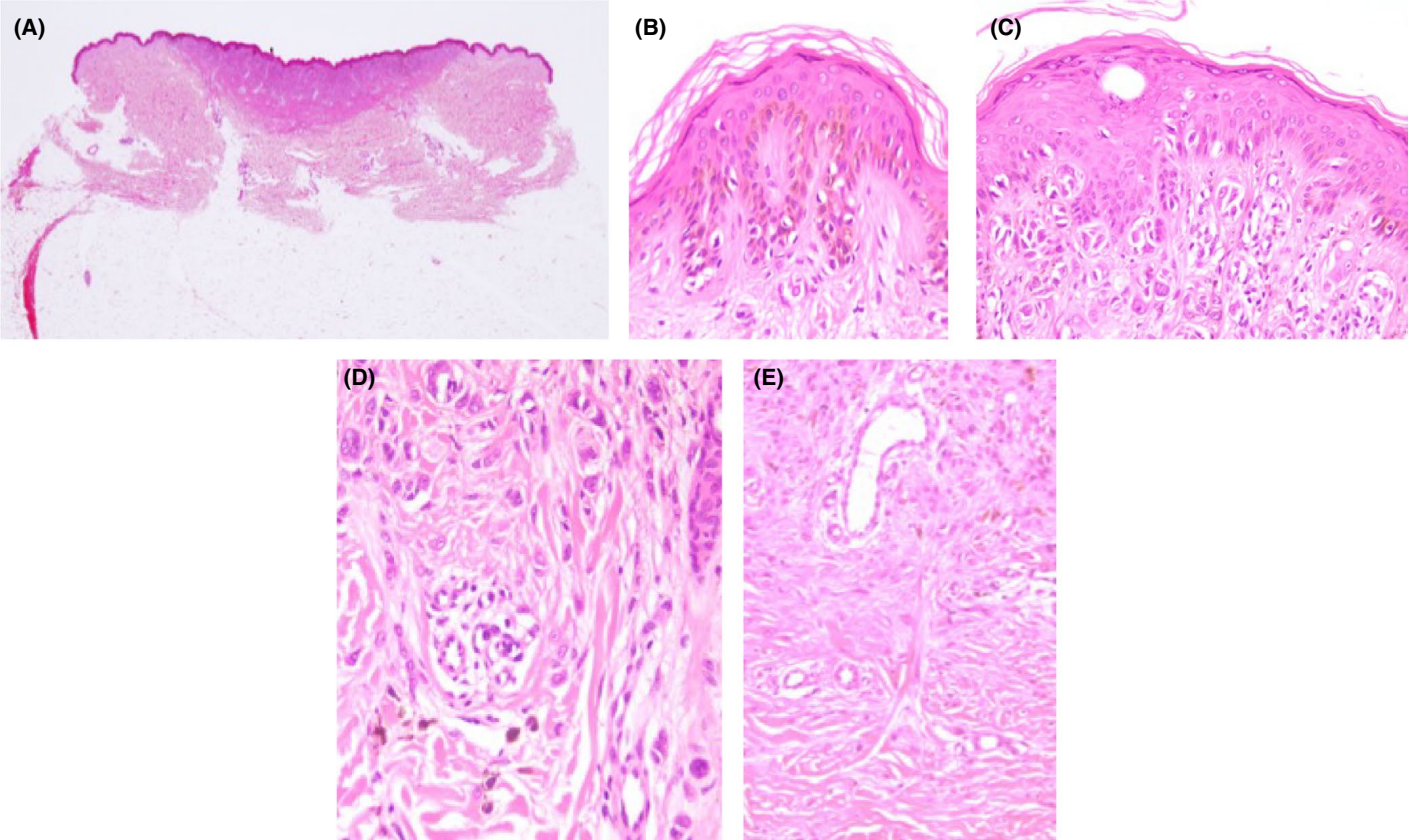
A‐E, Histopathological findings. A, The lesion showed an inverted triangle‐like form. B, Basal hypermelanosis and basilar proliferation of melanocytic cells at the lateral sides of the lesion. C, A small number of atypical melanocytes at the basal layer of the central portion of the lesion. Spindle‐shaped and epithelioid melanocytes were located at the dermoepidermal junction to in the upper dermis, partially forming nests. D, Smaller nests and single cells in the deeper dermis. (HE stain magnification ×200). E, Hyperplasia of collagenous fibers and capillary vessels was found around nevus cells. (HE stain magnification ×100)

Spitz nevus, a type of acquired melanocytic nevus,^1^ is common among children but rarely observed in adults.^1^ Under dermoscopy, it is characterized by a starburst pattern comprising a globular lesion and regular streaks in early lesions.^2^ Over a course of years, the streaks change into homogeneous patterns and gradually disappear.^2^ Histopathologically, Spitz nevus cells originate in the epidermis and gradually form alveolar foci at the basal layer of the epidermis and migrate into the dermis. In the course of progression, epidermal nevus cells disappear, leaving mature cells in the dermis.^3^ Although malignant melanoma was initially suspected, histopathological features, including only slight nuclear atypia, maturation of melanocytic cells, absence of necrosis, and fissures formed around the nests, finally exclude the possibility of malignancy.

Our case is unusual in dermoscopic features, such as central bluish‐white area and peripheral typical pigment network. Of 896 patients with Spitz nevus recently analyzed, only 14 (1.6%) had a bluish‐white veil, and 22 (2.5%) showed pigment network.^4^ The central bluish‐white area in our case should reflect a reduction in intraepidermal melanin levels and the presence of melanophages in the dermis. Moreover, the whitish region is thought to indicate fibrosis, and the pink structures are considered to be associated with the capillary hyperplasia of the upper dermis. On the other hand, typical pigment networks at the periphery should be linked to basal hypermelanosis and basilar proliferation of melanocytes on the lateral sides of the lesion. Interestingly, Colucci et al have reported an evolution of Spitz nevus with starburst pattern toward a lesion showing typical pigment network during its long course.^5^ Thus, unusual dermoscopic features in our case may reflect changes that unfold overtime. From another point of view, Moscarella et al investigated clinical, dermoscopic, and histopathological features of uncommon morphological variants of Spitz nevi and found that peripheral network and central bluish area or hyperpigmentation, features also seen in this case, were observed in desmoplastic type.^6^ Considering the presence of hyperplasia of collagenous fibers and unclear nests of nevus cells, the current case would also be interpreted as desmoplastic Spitz nevus showing such dermoscopic characteristics. Further studies are needed to elucidate how various unusual dermoscopic features develop in Spitz nevus.

## CONFLICT OF INTEREST

None.

## AUTHOR CONTRIBUTIONS

HK and HF: prepared manuscript. KH: assisted in the preparation of the manuscript. DF, MI, MM, and AK: performed surgery and followed the patient. MT: played a key role in the evaluation of dermoscopic features. TT: was involved in the diagnostic process, supervised, and reviewed manuscript. All authors have reviewed and approved the manuscript.
